# Biocompatibility in Ternary Fermentations With *Lachancea thermotolerans*, Other Non-*Saccharomyces* and *Saccharomyces cerevisiae* to Control pH and Improve the Sensory Profile of Wines From Warm Areas

**DOI:** 10.3389/fmicb.2021.656262

**Published:** 2021-04-29

**Authors:** Cristian Vaquero, Iris Loira, José María Heras, Francisco Carrau, Carmen González, Antonio Morata

**Affiliations:** ^1^EnotecUPM, Chemistry and Food Technology Department, ETSIAAB, Universidad Politécnica de Madrid, Madrid, Spain; ^2^Lallemand, Madrid, Spain; ^3^Área Enología y Biotecnología de Fermentaciones, Facultad de Química, Universidad de la Republica, Montevideo, Uruguay

**Keywords:** *Lachancea thermotolerans*, *Hanseniaspora vineae*, *Torulaspora delbrueckii*, *Metschnikowia pulcherrima*, acidity, freshness, biocompatibility, consortia strategies

## Abstract

Global warming is causing serious problems, especially, in warm regions, where musts with excess sugars and high pH produce wines with decreased freshness and unstable evolution. This study aimed to determine biocompatibility between yeast species, the capacity for microbiological acidification, and the aromatic profile produced in ternary fermentations in which *Lachancea thermotolerans* has been co-inoculated with *Hanseniaspora vineae*, *Torulaspora delbrueckii*, or *Metschnikowia pulcherrima*, and the fermentation process is subsequently completed with sequential inoculation of *Saccharomyces cerevisiae*. For this purpose, different cell culture media and instruments were used such as infrared spectroscopy, enzymatic autoanalyzer, chromatograph coupled with a flame ionization detector, spectrophotometric analysis, among others. The behavior of these yeasts was evaluated alone and in co-inoculation, always finishing the fermentation with sequential inoculation of *S. cerevisiae*, at a stable temperature of 16°C and with a low level of sulfites (25 mg/L) in white must. Significant results were obtained in terms of biocompatibility using population counts (CFU/ml) in differential plating media that permitted monitoring. Quantification of the five species was studied. Concerning acidification by *L. thermotolerans* in co-inoculations, we showed some metabolic interactions, such as the inhibition of acidification when *H. vineae*/*L. thermotolerans* were used, generating just over 0.13 g/L of lactic acid and, conversely, a synergistic effect when *M. pulcherrima*/*L. thermotolerans* were used, achieving 3.2 g/L of lactic acid and a reduction in pH of up to 0.33. A diminution in alcohol content higher than 0.6% v/v was observed in co-inoculation with the *L. thermotolerans*/*M. pulcherrima* yeasts, with total sugar consumption and very slow completion of fermentation in the inoculations with *H. vineae* and *T. delbrueckii*. The aromatic composition of the wines obtained was analyzed and a sensory evaluation conducted, and it was found that both *L. thermotolerans* and co-inoculations retained more aromatic esters over time and had a lower evolution toward the yellow tones typical of oxidation and that the best sensory evaluation was that of the Lt + Mp co-inoculation. *Lachancea thermotolerans* and co-inoculations produced wines with low levels of volatile acidity (<0.4 g/L). This work shows that good consortia strategies with binary and ternary fermentations of yeast strains can be a powerful bio-tool for producing more complex wines.

## Introduction

The fermentation of grape must is a complex process in which yeasts have great importance, because they generate the transformation of different sugars of the grape into ethanol, carbon dioxide, and thousands of metabolites, several of them with sensory repercussions. Historically, wine fermentations were spontaneous and the yeast population was dependent on the native microflora present in the vineyards and the cellars (Fleet, 2003). While sometimes producing more complexity, spontaneous fermentation is simultaneously more unpredictable and difficult to control in terms of quality and safety due to the variable yeast microbiota that can drive the process (Hierro et al., 2006; Carrau et al., 2020). Nowadays, although there is still a long way to go, it is increasingly being studied and understood how non-*Saccharomyces* yeasts, which normally do not finish the fermentation due to their low tolerance to ethanol and anaerobiosis or their specific nitrogen requirements (Monteiro and Bisson, 1991; Holm Hansen et al., 2001), generate many secondary metabolites that improve the organoleptic quality of the wine and its complexity. This is why it is interesting to combine these types of yeasts as starter cultures and carry out an in-depth study of their characteristics. It is becoming increasingly apparent that there is less and less genetic and phenotypic diversity in commercial strains of *S. cerevisiae* (Domizio et al., 2007; Carrau et al., 2015; Masneuf-Pomarede et al., 2016; Capozzi et al., 2020; Molinet and Cubillos, 2020). Therefore, non-*Saccharomyces* can be a powerful bio-tool to increase the diversity of flavors during wine fermentation and a potential strategy to mitigate both the development of spoilage microorganisms and the negative impacts of climate changes on wine quality (Berbegal et al., 2019) compared with traditional single fermentations with *S. cerevisiae* (Jolly et al., 2013). In our work, ternary fermentations refer to those fermentations in which three selected yeast species are inoculated to take advantage of the metabolic peculiarities of each one, just as in other works this term is used for fermentation with three different microorganisms (Fayyaz et al., 2020; Hashemi and Jafarpour, 2020), a management still poorly explored in the wine sector (Berbegal et al., 2020).

There is great interest in the use of multi-strain starters or consortia strategies with non-*Saccharomyces* yeasts to understand their effects on wine (Gschaedler, 2017; Perez et al., 2020). Within this group of yeasts, it has been seen how the yeast *L. thermotolerans* with a medium/high fermentative power (10% v/v) increases the acidity of the wine, lowers the pH, generates more esters, protects from oxidation (Morata et al., 2018, 2019c; Vaquero et al., 2020), and increases glycerol while slightly reducing the final alcohol content (Gobbi et al., 2013). *Hanseniaspora vineae* has a medium fermentative power (9–10% v/v; Martin et al., 2018) and medium adaptation to the increase of ethanol (Díaz-Montaño and de Jesús Ramírez Córdova, 2009), but a high contribution at the sensory level since it produces a large amount of aromatic compounds (Medina et al., 2013), such as 2-phenylethyl acetate and benzenoids, when used in monocultures or sequentially (Medina et al., 2013; Lleixà et al., 2016). *Torulaspora delbrueckii* is also widely used at an industrial level due to its low volatile acidity and contribution to wine fruitiness, structure (Jolly et al., 2013), and improvement in the texture of the wine (González-Royo et al., 2014; Medina-Trujillo et al., 2016). *Metschnikowia pulcherrima* has a low fermentative power (González et al., 2018); however, it produces a large quantity of terpenes, thiols, and esters, such as ethyl octanoate associated with the pear aroma (Clemente-Jimenez et al., 2004; Sadoudi et al., 2012), reduces the alcohol content (Contreras et al., 2014; Ciani et al., 2016), and can produce significant amounts of 2,6-dimethoxyphenol with a smoky aroma (Culleré et al., 2004; González-Royo et al., 2014). All these yeast species are increasingly being commercialized as starter cultures (Roudil et al., 2019).

A co-inoculation or sequential inoculation combines several advantages: the complexity provided by the non-*Saccharomyces* yeasts avoids the risk of spontaneous fermentations (Carrau et al., 2020), allows controlled fermentation (Viana et al., 2008; Ciani et al., 2010), and can generate exclusive wines. However, some disadvantages should be taken into account at winery scale, such as the occurrence of stuck fermentations due to competition for nutrients between *Saccharomyces* and non-*Saccharomyces* yeasts (Medina et al., 2012; Taillandier et al., 2014). The aim of this work was to analyze the biocompatibility of the following yeasts in binary (inoculum) or ternary (co-inoculum) fermentations, in all cases, finishing fermentation sequentially with *S. cerevisiae* for the production of high-quality wines in warm areas: *L. thermotolerans*, *H. vineae*, *T. delbrueckii*, and *M. pulcherrima*, with a strain of *S. cerevisiae* as the control.

## Materials and Methods

### Yeasts Used

The following non-*Saccharomyces* and *S. cerevisiae* yeast strains (also used in previous works) were used in the fermentations: the *H. vineae* (Hv205) yeast strain used in this study was isolated by Professor Francisco Carrau (Facultad de Química, Universidad de la República, Montevideo, Uruguay) and is currently under evaluation by “Oenobrands SAS, France,” *L. thermotolerans* (Lt) strain L31 (enotecUPM, ETSIAAB, UPM, Madrid, Spain; Morata et al., 2019b; Vaquero et al., 2020), *T. delbrueckii* (Td) strain Biodiva™TD291 (Lallemand Inc., Montreal, Canada; I. Loira et al., 2014; Nardi et al., 2019), *M. pulcherrima* (Mp) strain Flavia™MP346 (Lallemand Inc., Montreal, Canada; Bañuelos et al., 2016; Morata et al., 2019d), together with *S. cerevisiae* (Sc) strain 7VA (enotecUPM, ETSIAAB, UPM, Madrid, Spain).

### Fermentation Trials

**Figure 1:**
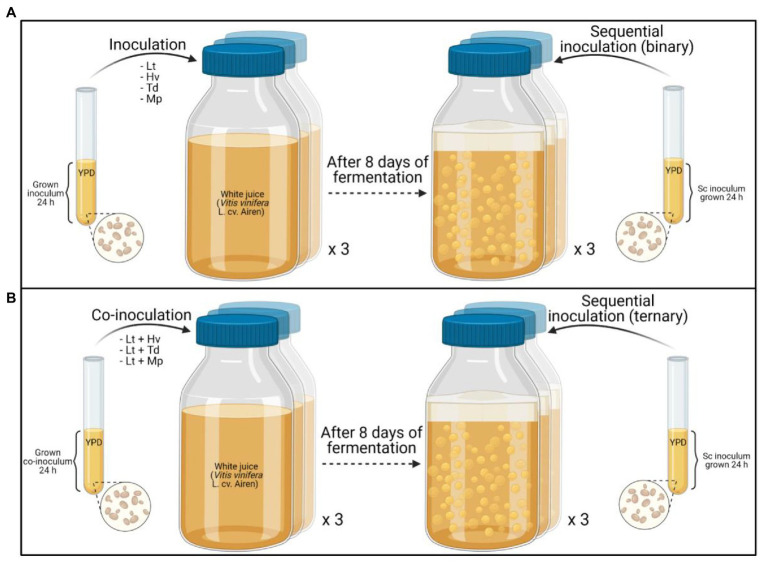
Graphical summary of the experimental plan: **(A)** sequential inoculation (binary), **(B)** sequential co-inoculation (ternary).

A single, medium-scale fermentation was carried out in triplicate using the four non-*Saccharomyces* yeasts plus the addition of *Saccharomyces* on day 8 of fermentation. The procedure was a single fermentation with Sc used as the control, sequential inoculations (binary) with Lt --> Sc, Hv --> Sc, Td --> Sc, Mp --> Sc (Figure 1A), and ternary fermentations with co-inoculations and sequential fermentations with Lt + Hv --> Sc, Lt + Td --> Sc, Lt + Mp --> Sc (Figure 1B) to evaluate the performance of the inoculum and co-inoculum in determining the fermentation kinetics and the fermentation power.

A white juice (*Vitis vinifera* L. cv. Airen) with a high pH (3.84), a sugar content of 196 g/L and a YAN of 237.4 mg/L were used. In the winery, the juice was obtained by pneumatic pressing and left to settle at a low temperature (4°C and 24 h) to clean it and reduce the initial microbial load. The 22,560 ml of must was pasteurized in 1 L ISO flasks (940 ml per flask) at 100°C for 10 min (to achieve minimum degradation of the must nutrients and sensory quality), using its own unsealed cap. Fermentations were performed in triplicate at 16°C with a total SO_2_ of 25 mg/L.

All yeast ferments were cultured in YPD (yeast extract, peptone, and dextrose agar; 10:20:20 g/L) with two sequential steps of 24 h each to homogenize the populations, and then 2% v/v was inoculated into the must. This inoculation ratio produced a final population of around 5-log CFU/ml. The inoculum with the lowest initial population was Hv with 0.6·10^5^ CFU/ml, followed by Lt with 1.2·10^5^ CFU/ml, Sc with 1.4·10^5^ CFU/ml, Td with 2.1·10^5^ CFU/ml and Mp with 2.2·10^5^ CFU/ml. Fermentations lasted for 31 days, and the basic oenological parameters were monitored daily using Fourier-transform infrared spectroscopy and enzymatic analysis. Wines were preserved with argon at the end of fermentation.

### Yeast Population Counts

Inoculated yeast populations were measured by plating of serial dilutions on YPD (yeast extract peptone, and dextrose agar; 10:20:20 g/L), YGC (yeast extract, glucose, and chloramphenicol agar; 5:20:0.2 g/L), lysine medium (Oxoid, Hampshire, UK), and CHROMagar™ Candida (Conda, Barcelona, Spain). Samples were taken from the fermentations on days 2, 4, 6, and 8, and on day 8, after the samples were taken, the *Saccharomyces* was inoculated. Lysine medium is selective for non-*Saccharomyces* and species identification was done in a differential chromogenic medium. With this method, a rigorous count was achieved in the co-inoculations between the *L. thermotolerans* yeast and the other three non-*Saccharomyces* yeasts.

### Oenological Parameters by Infrared Spectroscopy

The equipment OenoFoss (FOSS Iberia, Barcelona, Spain) using FTIR was used to identify and quantify major compounds such as residual sugars, organic acids, total acidity (as tartaric acid), and volatile acidity (as acetic acid). This technique also estimates pH value.

### pH Determination

The pH of each sample was measured with a Crison micropH 2000 pHmeter (HACH LANGE, Barcelona, Spain).

### Analysis of Lactic Acid

Lactic acid was measured enzymatically using an Y15 enzymatic autoanalyzer (Biosystems, Barcelona, Spain).

### Color Parameters Analyzed by UV-Visible Spectrophotometry

The absorbance at 420 nm was determined using an Agilent 8,453 spectrophotometer (Agilent Technologies S.L., Madrid, Spain) and a 1-mm path length glass cuvette.

### Analysis of Fermentative Volatile Compounds Using GC-FID

An Agilent Technologies 6,850 gas chromatograph (Network GC System) coupled to a flame ionization detector (GC-FID) was used for this analysis as described in Abalos et al. (2011) and Loira et al. (2013). Samples were injected after filtration through 0.45 μm cellulose methyl ester membrane filters (Phenomenex, Madrid, Spain). The injector temperature was 250°C, and the detector temperature was 300°C. The column used was a DB-624 column (60 m × 250 μm × 1.40 μm) with 1:10 split flow, calibrated with the following compounds as external standards: acetaldehyde, methanol, 1-propanol, 1-butanol, 2-butanol, isobutanol, 2-methyl-1-butanol, 3-methyl-1-butanol, 2-phenylethyl acetate, 2-phenylethyl alcohol, diacetyl, ethyl acetate, isoamyl acetate, isobutyl acetate, ethyl butyrate, 3-ethoxy-1-propanol, ethyl lactate, and hexanol. 4-methyl-2-pentanol was used as the internal standard (all compounds from Fluka, Sigma-Aldrich Corp., Buchs SG, Switzerland). The temperature ramp of the method was 40°C for the first 5 min, followed by a linear increase of 10°C per minute up to 250°C. This temperature was maintained for 5 min. The total run time for each sample was 40 min. The carrier gas used was hydrogen with a column flow rate of 2.2 ml min^−1^, and 100 μl of the internal standard (4-methyl-2-pentanol, 500 mg/L; Fluka Chemie GmbH, Buchs, Switzerland) was added to the 1 ml test samples. The detection limit was 0.1 mg/L. The volatile compounds analyzed with this technique were precalibrated with five-point calibration curves (*r*^2^), and all compounds had an *r*^2^ > 0.999, except 2,3-butanediol (0.991) and phenylethyl alcohol (0.994).

### Sensory Analysis

A panel of eight experienced tasters (aged between 30 and 65 years, two women and six men) evaluated the wines that had been bottled and kept under refrigeration for 3 months. The blind tasting took place in the tasting room of the Department of Chemistry and Food Technology of the Polytechnic University of Madrid, which was equipped with fluorescent lighting, and the samples were presented in random order. The wines (30 ml/tasting glass) were served at 12 ± 2°C in standard, odorless tasting glasses. A glass of water was also provided to the panelists for cleaning the palate between samples. Before the generation of a consistent terminology by consensus, three visual attributes, seven for aroma, and four for taste were chosen to describe the wines. Panelists used a scale of 1–5 to rate the intensity of each attribute. On the scale, 1 represented “non-perceptible attribute” and 5 represented “strongly perceptible attribute.” Each panelist also evaluated the overall impression, taking into account olfactory and gustatory aspects, as well as the lack of defects. The tasting sheets also had a final blank space for any additional comments or observations on sensory notes or nuances not previously included as attributes.

### Statistical Analysis

Statgraphics v.5 software (Graphics Software Systems, Rockville, MD, United States) was used to calculate mean, standard deviation, analysis of variance (ANOVA), and least significant difference (LSD). Significance was set at *p* < 0.05 for the ANOVA matrix *F* value on the results of the sensory analysis. All treatments were evaluated in triplicate.

## Results

### Counting of Viable Microbes in Binary (Inoculations) and Ternary (Co-Inoculations) Fermentations

**Figure 2:**
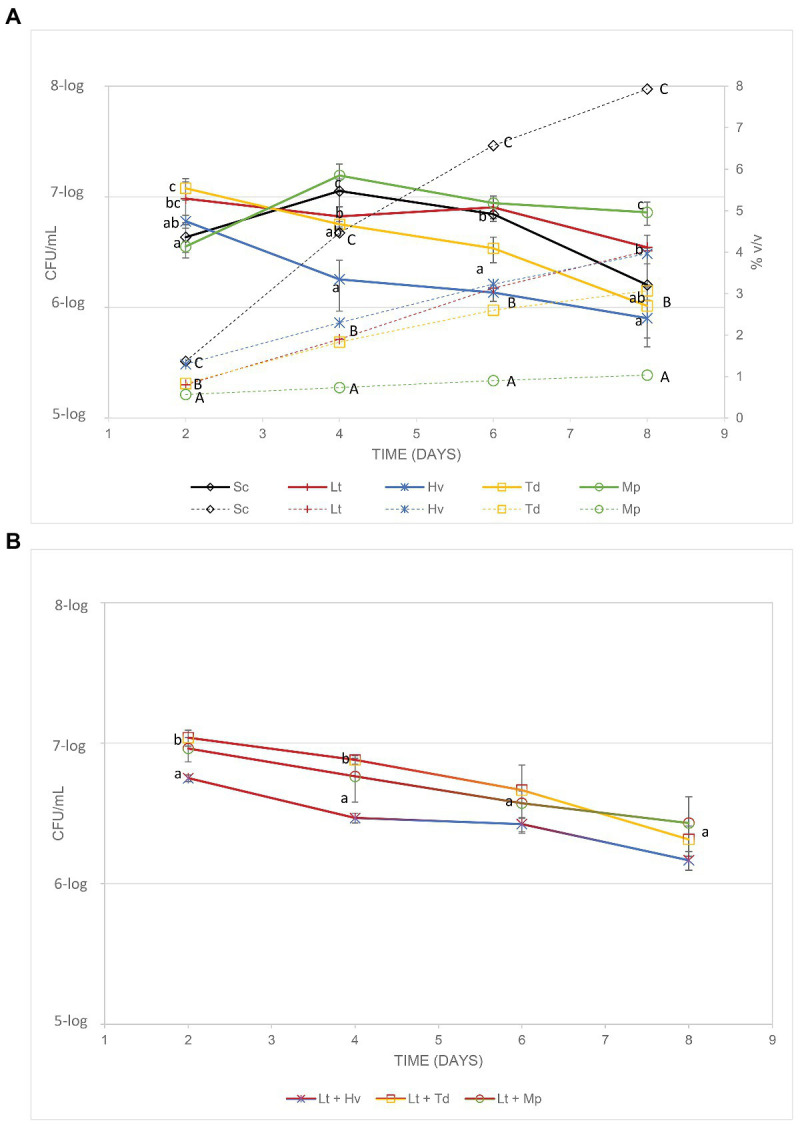
**(A)** Lines show the cell count of each co-inoculation (CFU/ml) and the dotted lines show the increase in alcohol content of the various fermentations. **(B)** Cell counts of the yeasts (CFU/ml) involved in the co-inoculations.

The yeast population and its evolution during fermentation (Figure 2A) were monitored until day 8 through the different culture media (YPD, YGC, Lysine, and CHROMagar™ Candida), just before inoculation of *S. cerevisiae*. The effectiveness of the pasteurization was tested in three uninoculated controls that remained unfermented during all the experiments. *Saccharomyces* and non-*Saccharomyces* were inoculated at a similar population for all the yeast species (6-log CFU/ml). On day 2, all fermenters were above 6.5-log CFU/ml. On day 4, binary inoculations of the Mp and Sc yeasts had their largest population, Td and Lt had maintained the same levels, and the Hv colony count had decreased. On day 6, the Lt, Sc, and Mp yeasts had maintained the same levels while the Hv and Td yeasts had continued to decline. On the last day of the count (day 8), all the yeasts had declined in population, except Mp, the levels of which remained similar even though Mp showed the lowest alcohol content that day. In ternary co-inoculations, it was observed that, except for the co-inoculation of Lt + Hv, the treatments had a population of 7-log CFU/ml. A count of the population of these co-inoculations was also carried out (Figure 2B), showing a progressive drop in the population as the alcoholic strength increased.

**Figure 3:**
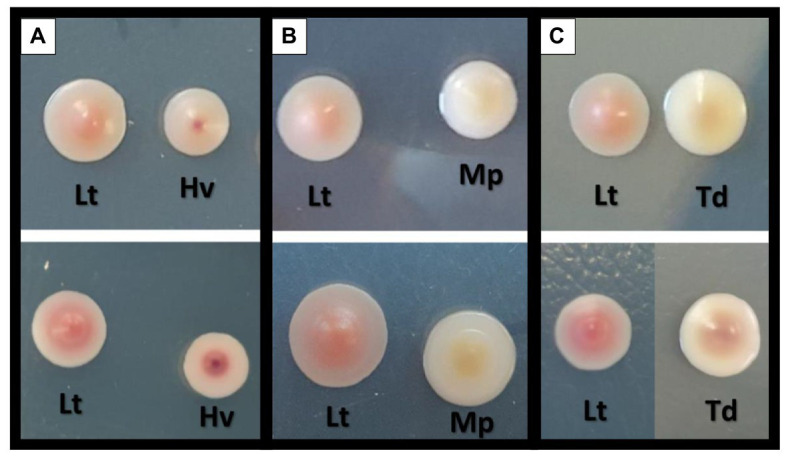
Comparison and colorimetric evolution of the different yeasts on two different days with the culture medium of CHROMagar™ Candida. The top of the figure shows the yeasts on the 4th day of growth and the bottom of the figure shows the yeasts on the 7th day of growth. **(A)** *L. thermotolerans* (Lt) with *H. vineae* (Hv). **(B)** *L. thermotolerans* (Lt) with *M. pulcherrima* (Mp). **(C)** *L. thermotolerans* (Lt) with *T. delbrueckii* (Td).

Photos of the isolated yeast colonies were taken to observe the differences in color of the co-inoculates used. Pictures were taken on the 4th and 7th days of growth in CHROMagar™ Candida culture medium, and an evolution in the color of all the yeasts was observed (Figure 3). The Lt yeast present in all the co-inoculations was observed to have a progressive pink color that was less intense toward the colony’s periphery. In the Hv yeast, there was a slightly smaller colony size than that of the Lt with a marked red spot in the center that increased in intensity and size with the passing of the days (Figure 3A). The Mp yeast began with a creamy white color; eventually, an intense yellow color was observed in its center, fading toward the periphery. It was always smaller than the Lt colony (Figure 3B). The Td yeast, which in its first days of growth presented a creamy appearance and a slightly larger size than the Lt, showed an evolution toward brown tones in its center (Figure 3C).

### Biocompatibility Between Yeasts

**Figure 4:**
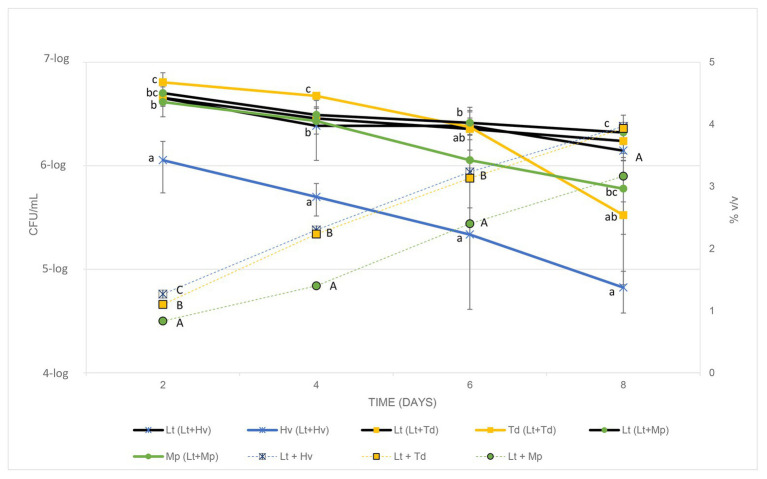
Cell counts of each yeast in the co-inoculations (CFU/ml) and the increase in alcohol content of the different co-inoculations for 8 days, testing every 2 days.

In co-inoculations, it is important to understand how yeasts behave when they compete with each other. For this reason, both yeasts were counted in the three ternary co-inoculations. The Lt yeast had a strong and regular fermentation, as can be seen in Figure 4, during the 4 days of counts, quite the opposite of the Hv, which started its fermentation more weakly with a smaller yeast population that did not manage to increase. The Td yeast underwent fermentation with a higher population count and a gradual decline, but it was not until the 6th day that it began to decline further, this being severe on the 8th day. The Mp yeast showed a regular decrease in population. With these results, it can be seen that the best biocompetitor is the Lt yeast compared to the other non-*Saccharomyces*.

### Oenological Parameters

**Figure 5:**
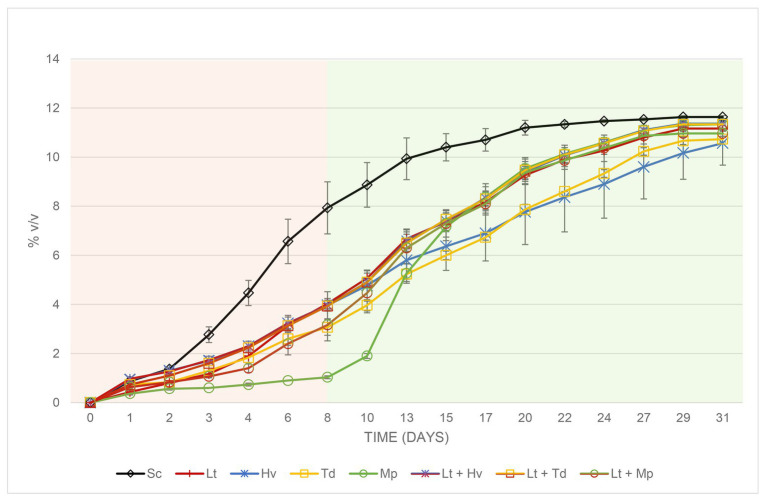
Evolution of the alcoholic strength with its error bars produced during the different binary (inoculum) and ternary (co-inoculum) fermentations. The two colors in the figure show the fermentation before and after inoculation of the Sc yeast.

In the fermentations with Sc (Figure 5), a faster sugar metabolization and ethanol formation were observed, reaching 7.93 ± 1.06% v/v on the 8th day, in contrast to the slow fermentation of the inoculation with Mp with an alcoholic degree of 1.03 ± 0.06% v/v on the same day. The other species showed a moderate fermentation rate. From the inoculation of the Sc on day 8, all the fermentations evolved until sugar depletion before day 31, except the fermentations with the Hv and Td inoculations. They were compared, and it was observed that on day 31 of fermentation, the difference in alcoholic strength between Sc and Mp binary inoculations was 0.30 ± 0.00% v/v (Figure 5) and the difference between Sc and the Lt + Mp ternary co-inoculation was 0.67 ± 0.06% v/v, always lower when using Mp.

Table 1 shows that on day 8 all the yeasts, except the Mp inoculum, which was added later, and the Sc inoculum, which was added earlier, were between 3 and 4% v/v. On the other hand, Table 2 shows a higher total acidity in the co-inoculation of Lt + Mp compared to the other treatments, even higher than the pure inoculation of Lt. In contrast, the inoculation with Td had a lower total acidity concentration compared to the other treatments. In addition, all the yeast treatments had a volatile acidity of under 0.35 g/L. As already mentioned, both Td and Hv inoculations had slower fermentation profiles compared to the other inoculations, which might be due to a lack of certain vitamins or amino acids; this demands further study. The Td inoculum needed 36 days to finish fermentation with 11.5 ± 0.06% v/v and 4.13 ± 1.33 g/L of residual glucose/fructose, while the Hv inoculum needed 62 days with 11.7 ± 0.01% v/v with 0.40 ± 0.10 g/L of residual glucose/fructose.

**Table 1:** Oenological parameters of the different fermentations on day 8 before the final inoculation with Sc.

Parameters	Ethanol (%v/v)	Total acidity (g/L)	Volatile acidity (g/L)	Glucose/Fructose (g/L)	pH
YEAST/DAYS	8	8	8	8	8
Sc	7.93 ± 1.06d	3.13 ± 0.06c	0.31 ± 0.07bcd	61.57 ± 16.64a	3.66 ± 0.03c
Lt	4.03 ± 0.21c	3.90 ± 0.17d	0.27 ± 0.05abc	118.73 ± 3.86b	3.54 ± 0.02b
Hv	3.97 ± 0.55c	2.80 ± 0.00ab	0.34 ± 0.01d	128.27 ± 8.22bc	3.79 ± 0.01d
Td	3.07 ± 0.32b	2.67 ± 0.06a	0.24 ± 0.01a	140.63 ± 5.18c	3.80 ± 0.01d
Mp	1.03 ± 0.06a	2.73 ± 0.06ab	0.29 ± 0.01abcd	174.93 ± 1.50d	3.82 ± 0.01d
Lt + Hv	3.97 ± 0.21c	2.90 ± 0.10abc	0.31 ± 0.00cd	124.87 ± 3.81b	3.77 ± 0.01d
Lt + Td	3.93 ± 0.21bc	3.00 ± 0.10bc	0.25 ± 0.00ab	124.73 ± 2.60b	3.71 ± 0.02c
Lt + Mp	3.17 ± 0.65bc	4.30 ± 0.46e	0.33 ± 0.03d	132.23 ± 10.33bc	3.48 ± 0.08a

Values are means with standard deviations, *n* = 3. Values with the same letter in the same column are not significantly different (*p* < 0.05).

**Table 2:** The last day of the analysis (day 31).

Parameters	Ethanol (%v/v)	Total acidity (g/L)	Volatile acidity (g/L)	Glucose/Fructose (g/L)	pH
Yeast/Days	31	31	31	31	31
Sc	11.63 ± 0.06d	2.83 ± 0.12bc	0.26 ± 0.06b	0.00 ± 0.00a	3.75 ± 0.01d
Lt	11.17 ± 0.12bcd	4.20 ± 0.20e	0.23 ± 0.03ab	1.33 ± 2.31a	3.51 ± 0.02b
Hv	10.57 ± 0.90a	2.63 ± 0.06ab	0.33 ± 0.03c	27.97 ± 18.35c	3.82 ± 0.01e
Td	10.73 ± 0.12ab	2.53 ± 0.06a	0.19 ± 0.01a	13.87 ± 0.15b	3.80 ± 0.01e
Mp	11.33 ± 0.06cd	3.00 ± 0.00cd	0.29 ± 0.02bc	3.00 ± 2.65ab	3.70 ± 0.00c
Lt + Hv	11.37 ± 0.06cd	3.10 ± 0.10cd	0.25 ± 0.02b	2.33 ± 2.31a	3.72 ± 0.03cd
Lt + Td	11.33 ± 0.06 cd	3.17 ± 0.06d	0.17 ± 0.01a	1.33 ± 0.58a	3.70 ± 0.01c
Lt + Mp	10.97 ± 0.12abc	4.73 ± 0.35f	0.28 ± 0.06bc	0.00 ± 0.00a	3.42 ± 0.07a

Values are means with standard deviations, *n* = 3. Values with the same letter in the same column are not significantly different (*p* < 0.05).

### Lactic Acidity and pH

**Figure 6:**
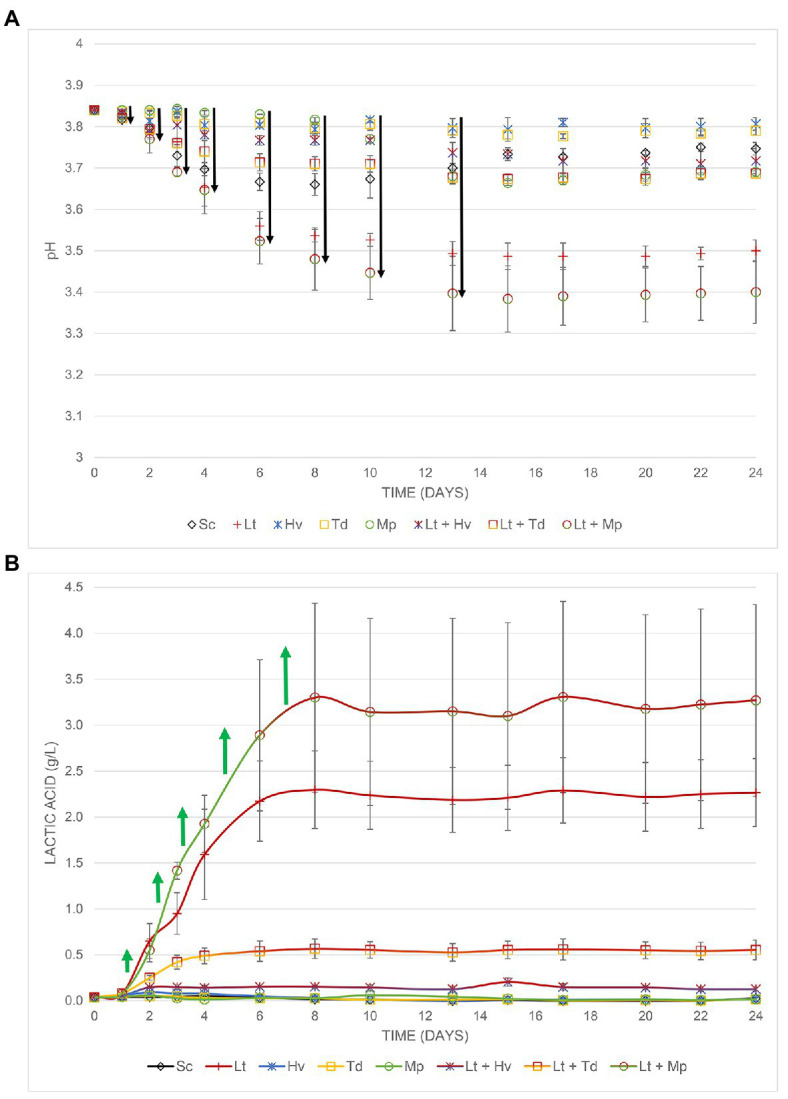
**(A)** pH decrease, **(B)** Increase in lactic acid from the different fermentations both with their error bars.

In this trial, it was shown how the Lt yeast interacts with three other non-*Saccharomyces* yeasts (Figure 6). Fermentation (Figure 6A) started with a must that had a pH of 3.84 and ended with 3.75 ± 0.01 in the Sc yeast. The inoculum of Lt reduced the pH by 0.24 ± 0.02 g/L, while the co-inoculum of Lt + Mp reduced the pH by 0.33 ± 0.07 g/L. It was observed (Figure 6B) that the co-inoculation of Hv + Lt inhibited the formation of this acid, producing only 0.13 ± 0.03 g/L, despite having a smaller population of Hv yeast during the whole process. It was seen that the co-inoculation of Td + Lt did not produce the same amount of lactic acid as the Lt yeast either, producing 0.55 ± 0.11 g/L, possibly because during the first 6 days, there was a larger population of Td yeast. Finally, the co-inoculation of Lt + Mp greatly favored the production of lactic acid, producing 3.27 ± 1.04 g/L. The value was even higher than that of the Lt inoculum, which suggests a synergistic behavior.

### Fermentative Volatiles

**Figure 7:**
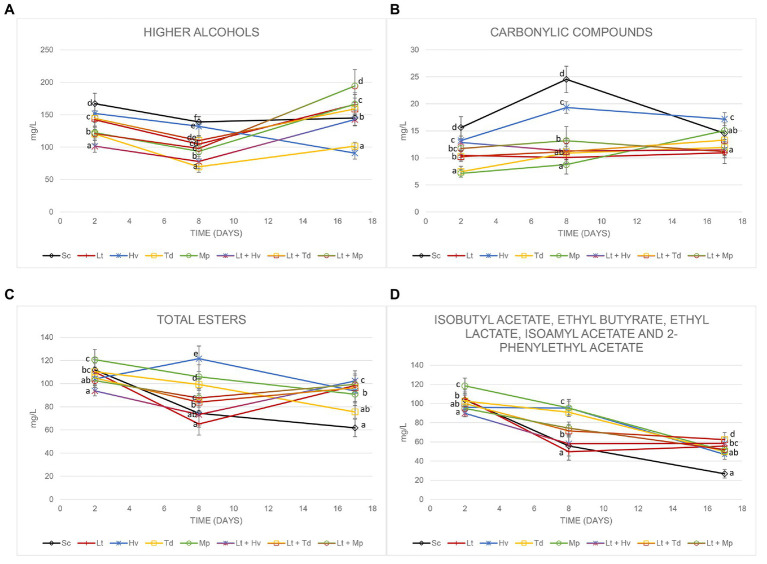
Concentration of **(A)** higher alcohols, **(B)** carbonyl compounds, **(C)** total esters, and **(D)** isobutyl acetate, ethyl butyrate, ethyl lactate, isoamyl acetate, and 2-phenylethyl acetate on days 2, 8, and 17 of fermentation. Values are means ± SD (*n* = 3). A different letter for the same day means significant differences (*p* < 0.05).

To evaluate the impact of these non-*Saccharomyces* yeasts, we focused on four different categories: higher alcohols, carbonyl compounds, total esters, and positive aromatic esters. The analyses were carried out on days 2, 8, and 17 to evaluate their evolution. The higher alcohols (Figure 7A) on day 17 of fermentation were all below 300 mg/L. The highest content was achieved in the fermentation by the co-inoculum Lt + Mp with 194.38 ± 26.59 mg/L, and the lowest value was obtained by the inoculum of Hv with 90.71 ± 9.22 mg/L, with isobutanol being the major compound at the beginning of fermentation and 3-methyl-1-butanol on day 17 of fermentation (Table 3). It was observed that the production of 1-propanol was always higher at the end of the fermentations in inoculations with Lt.

**Table 3:** Concentration (mg/L) of the different volatile compounds analyzed on day 17 of fermentation in the different binary inoculations and ternary co-inoculations.

Compound/Yeast	Sc	Lt --> Sc	Hv --> Sc	Td --> Sc	Mp --> Sc	Lt + Hv --> Sc	Lt + Td --> Sc	Lt + Mp --> Sc
Propan-1-ol	32.30 ± 2.62de	30.95 ± 2.22cd	9.21 ± 1.27a	14.59 ± 1.64a	23.13 ± 3.53b	23.44 ± 2.93b	25.62 ± 1.71bc	36.96 ± 6.64e
Diacetyl	4.62 ± 0.96de	1.61 ± 0.08a	2.04 ± 0.38ab	2.16 ± 0.50ab	5.42 ± 1.31e	4.14 ± 0.24cd	3.14 ± 0.53bc	3.02 ± 0.38bc
Ethyl acetate	27.68 ± 2.17b	36.18 ± 0.93d	34.93 ± 2.81cd	18.42 ± 2.09a	30.19 ± 4.15bc	34.46 ± 1.16cd	26.69 ± 3.32b	38.35 ± 4.12d
Butan-2-ol	0.00 ± 0.00a	0.00 ± 0.00a	0.00 ± 0.00a	2.79 ± 0.10b	0.00 ± 0.00a	0.00 ± 0.00a	0.00 ± 0.00a	0.00 ± 0.00a
Isobutanol	14.85 ± 3.55ab	22.72 ± 4.36c	13.12 ± 1.98ab	12.03 ± 1.98a	33.45 ± 3.61d	18.17 ± 0.30bc	32.59 ± 4.59d	36.85 ± 1.70d
Butan-1-ol	1.32 ± 2.29ab	4.02 ± 0.25c	3.97 ± 0.19c	0.00 ± 0.00a	2.71 ± 2.35bc	3.72 ± 0.17c	4.23 ± 0.54c	4.12 ± 0.25c
Acetoin	9.92 ± 0.98bc	9.34 ± 0.88bc	15.14 ± 0.82d	9.78 ± 1.02bc	9.63 ± 0.74bc	7.35 ± 1.20a	10.16 ± 0.23c	8.13 ± 1.82ab
3-Methyl-1-butanol	53.13 ± 2.19bc	60.94 ± 3.61cd	41.38 ± 3.80a	42.87 ± 1.56a	61.14 ± 3.49d	43.87 ± 1.63a	53.51 ± 6.93bc	56.19 ± 7.08bcd
2-Methyl-1-butanol	25.55 ± 0.71bc	23.69 ± 3.33b	10.91 ± 1.70a	13.81 ± 0.35a	23.54 ± 2.61b	29.31 ± 2.29c	21.47 ± 0.69b	37.76 ± 6.05d
Isobutyl acetate	1.95 ± 0.30bcd	2.86 ± 0.96d	1.75 ± 0.36bc	1.68 ± 0.28bc	1.42 ± 0.33b	2.39 ± 0.89cd	1.73 ± 0.56bc	0.00 ± 0.00a
Ethyl butyrate	1.32 ± 0.05b	0.00 ± 0.00a	0.00 ± 0.00a	0.00 ± 0.00a	1,73 ± 0,45c	0.00 ± 0.00a	0.00 ± 0.00a	0.00 ± 0.00a
Ethyl lactate	7.31 ± 0.99ab	6.60 ± 0.70a	11.81 ± 2.33d	7.29 ± 0.88ab	10.05 ± 1.22 cd	9.37 ± 1.80bc	7.31 ± 1.23ab	9.05 ± 0.50bc
Isoamyl acetate	2.01 ± 0.25b	0.00 ± 0.00a	0.00 ± 0.00a	0.00 ± 0.00a	0.00 ± 0.00a	0.00 ± 0.00a	0.00 ± 0.00a	0.00 ± 0.00a
2-phenylethyl alcohol	17.82 ± 0.87b	23.48 ± 1.47c	12.16 ± 0.27a	15.36 ± 0.43b	22.31 ± 1.97c	23.98 ± 1.45c	21.52 ± 2.20c	22.49 ± 3.46c
2-phenyl ethyl acetate	21.48 ± 3.83a	52.76 ± 1.43cd	45.16 ± 4.72b	48.17 ± 2.31bc	47.45 ± 3.58bc	56.14 ± 4.81de	60.66 ± 6.89e	51.95 ± 3.42bcd

Values are means with standard deviations, *n* = 3. Values with the same letter in the same row are not significantly different (*p* < 0.05).

There was a great fluctuation of carbonylic compounds (Figure 7B), including acetoin and diacetyl, in the tumultuous phase of fermentations. A slight increase in diacetyl and a decrease in acetoin were observed during fermentations. The Mp inoculum always produced an increase in its carbonylic compounds, starting with 7.14 ± 0.71 mg/L and reaching 15.05 ± 2.05 mg/L on day 17. It was observed that the diacetyl at the end of the fermentation was around 3 mg/L.

Total esters (Figure 7C), which include ethyl acetate, isobutyl acetate, ethyl butyrate, ethyl lactate, isoamyl acetate, and 2-phenylethyl acetate, also showed slight fluctuations on day 8. The largest amount of ethyl acetate (Table 3) was produced by the co-inoculum Lt + Mp with 38.35 ± 4.12 mg/L on day 17; however, on day 8 of fermentation the average ethyl acetate production in the fermentations with the Lt inoculum was 14.04 ± 1.10 mg/L. Figure 7D excludes ethyl acetate, because it is related to undesirable aromas, although on day 17 none of the fermentations had exceeded 40 mg/L. It was seen that all the fermentations with Lt showed stabilization and a greater quantity of these aromatic esters. A production of between 7 and 12 mg/L was obtained from ethyl lactate during fermentation. Isoamyl acetate was detected in small quantities in all fermentations and isobutyl acetate was hardly detected.

**Figure 8:**
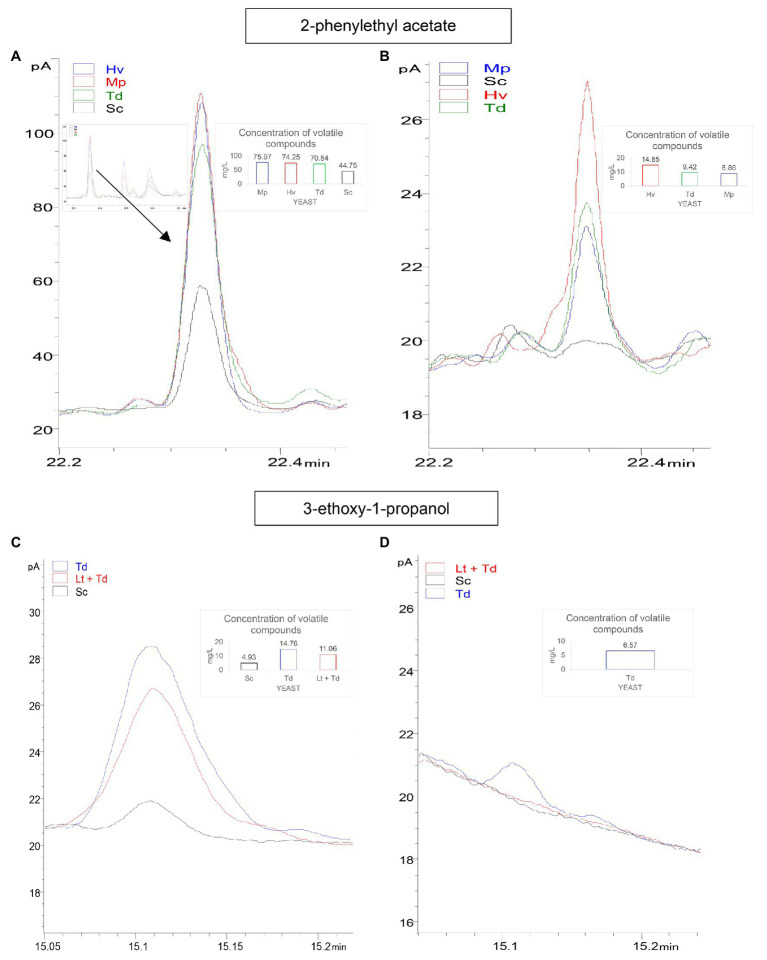
In this figure, concentration variation of two important aroma compounds for the different treatments is shown. **(A)** GC-FID chromatograms of: 2-phenylethyl acetate (day 8 of fermentation); the black line is Sc yeast, the green line is Td inoculum, the red line is Hv inoculum, and the blue line is Mp inoculum. **(B)** 2-phenylethyl acetate (day 31 of fermentation). **(C)** 3-ethoxy-1-propanol; black line is Sc yeast, the red line is Lt + Td co-inoculum, and the blue line is Td inoculum. **(D)** 3-ethoxy-1-propanol.

Due to the importance of 2-phenylethyl acetate, a final check of the amount of this compound was carried out on day 31 of fermentation. The results showed that the inoculations of Hv, Td and Mp had a high content of this compound, corresponding to 74.25 ± 4.77, 70.84 ± 1.07, and 75.97 ± 6.89 mg/L, respectively, on day 8 before inoculating Sc to complete fermentation, compared to the Sc yeast, which was 44.75 ± 8.96 mg/L (Figure 8A). This amount decreased until day 31 of fermentation with results of 14.85 ± 2.48, 9.42 ± 0.72, and 8.86 ± 0.83 mg/L, respectively (Figure 8B).

On the other hand, 3-ethoxy-1-propanol was also analyzed on day 31 of fermentation. It was observed on day 8 of fermentation before inoculating Sc that this compound was 4.93 ± 0.41 in the Sc inoculum, 14.76 ± 0.74 in the Td inoculum, and 11.06 ± 0.36 mg/L in the co-inoculum Lt + Td (Figure 8C), while on day 31 of fermentation, it only appeared in the inoculum Td at 6.57 ± 0.34 mg/L (Figure 8D).

### Color

**Figure 9:**
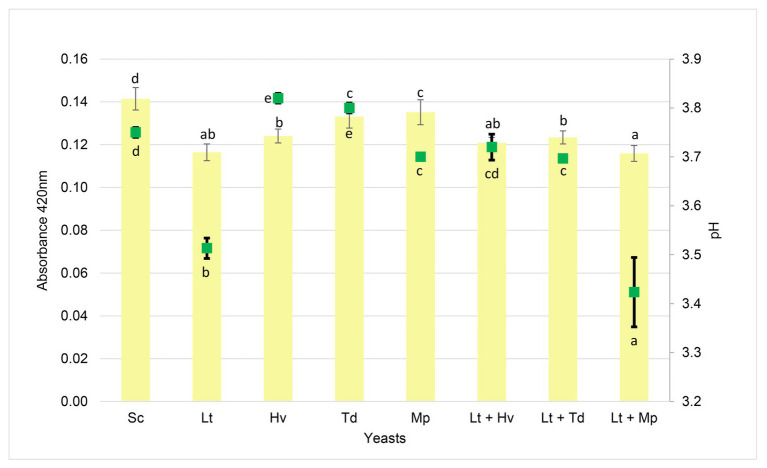
Absorbance at 420 nm (yellow color bars) by the wine samples and pH (green squares) at the end of each fermentation. Values are means ± SD (*n* = 3). A different letter for the same parameter (pH or absorbance) indicates significant differences (*p* < 0.05).

A spectrophotometric analysis was carried out at 420 nm (Figure 9) to evaluate this color parameter. An expected trend was observed in which, at a lower pH, the absorbance at 420 nm was lower in the inoculation and co-inoculations with the Lt. There was a correlation between the inoculation and co-inoculations of the Lt, giving a result of 0.92, compared to the negative correlation of the other inoculations with a result of −0.65.

### Sensory Analysis

**Figure 10:**
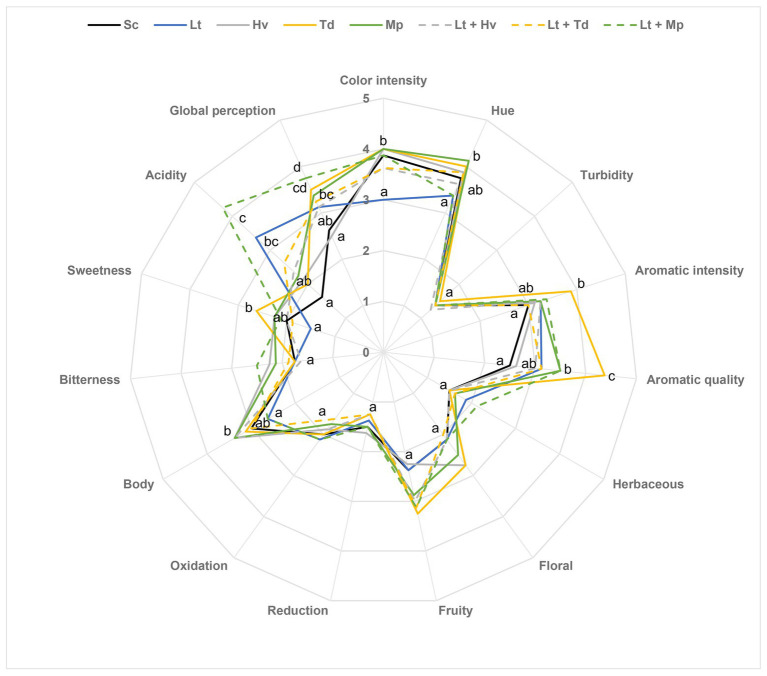
Sensory analysis of medium-scale wines. The values are the average from eight tasters. The same attributes with the same letter are not significantly different (*p* < 0.05).

A sensory analysis of the wines was done to assess the expressiveness of the yeasts (Figure 10). In general, the Sc control was the most neutral wine with no significant points. Significant differences were observed in the color intensity and hue parameter, with the Lt inoculum producing less golden tones. A significant difference was also perceived in both the intensity and quality of aroma in the Td inoculum. There were no significant differences in the floral and fruit aroma, but in general, there were more fruit aromas than floral aromas. In terms of wine body, the inoculations with Hv and Mp and the co-inoculum of Lt + Hv elicited the highest value. Although there were no significant differences in bitterness, the group of tasters did detect slightly more bitterness in the Lt + Mp co-inoculum. Concerning sweetness, the Td inoculum elicited the highest value, probably because it was the yeast that had the most residual sugars at around 5 g/L. In reference to the acidity of the wines, data obtained in the enzymatic autoanalyzer were confirmed in the sensory analysis. The Lt inoculum and Lt + Mp co-inoculum showed the most significant differences. Finally, in the global perception parameter, the Lt + Mp co-inoculum and Td inoculum were considered the best.

## Discussion

It has been studied how mixed cultures as yeast starters for grape must fermentation can generate wines with higher amounts of metabolites than single cultures (Gschaedler, 2017) although there may be some variations on a major scale. However, a good inoculation or co-inoculation with competitive strains and with populations higher than 7-log CFU/ml can generate competitiveness in the 1st day of fermentation when non-*Saccharomyces* yeasts make their greatest contribution of metabolites, provided that the population of wild yeasts is lower than 5-log CFU/ml, which is possible with quick harvest, transport, and set-up in the winery at the lowest possible temperature (Comitini et al., 2011; Maturano et al., 2012; Gobbi et al., 2013; Tristezza et al., 2016; Dutraive et al., 2019). In addition, non-thermal technologies, such as Ultra High Pressure Homogenization (UHPH), pulsed electric fields (PEFs), and pulsed light (PL), which totally or partially eliminate the wild microbial load for more controlled fermentations with less SO_2_ addition, are increasingly being investigated (Morata et al., 2019a, 2020; Santamera et al., 2020).

In the present study, we wanted to study the biocompatibility of non*-Saccharomyces* species in more depth by carrying out population counts in differential plating media to monitor the implantation quality of each yeast. It was observed that in the co-inocula, the Lt yeast lived longer than the rest of the non-*Saccharomyces*. On the other hand, in the images taken, it is clearly seen that the Lt yeast obtained its characteristic deep pink color in agreement with our previous work (Morata et al., 2020), and that the Mp yeast had an orange color beneath the colony due to the pulcherrimin pigment, which contains iron (Türkel and Ener, 2009; Sipiczki, 2020).

Among the oenological parameters, it is worth mentioning the slow fermentation of the binary inoculation of Hv inoculation, which may be due to the exhaustion of different vitamins, including thiamine, which hinders the fermentation of Sc (Bataillon et al., 1996; Martin et al., 2018). It is also important to add that there are certain strains of Mp capable of reducing ethanol (between 0.9 and 1.6% v/v) when co-inoculated with Sc (Gonzalez et al., 2013; Jolly et al., 2013), which was observed to a slight extent in our fermentations. In addition, all yeasts had a volatile acidity <0.35 g/L, with the Td binary inoculation and ternary co-inoculation having the lowest values, as seen in other studies (Renault et al., 2009; Canonico et al., 2016; Tondini et al., 2019).

It should also be noted that the Lt yeast is capable of producing large amounts of lactic acid depending on different factors (Binati et al., 2019; Vaquero et al., 2020). The greatest amount of lactic acid was produced around day 6, as shown in previous studies (Morata et al., 2019b; Vaquero et al., 2020). It is worth noting the great synergy between the Lt + Mp ternary co-inoculation yeast for lactic acid production and, in contrast, the low production with the ternary co-inoculation of Lt + Hv, possibly due to inhibition or lack of nutrients. To increase lactic acid production, a sequential inoculation between day 4 and day 6 would potentially be recommended after the Lt inoculum has generated its maximum amount of lactic acid and the alcohol is still insufficient to damage the growth of the other non-*Saccharomyces*.

In reference to volatile compounds, they are a large and interesting group that, thanks to non-*Saccharomyces* yeasts, can influence the aromatic profile of the wine. Some of these yeasts can survive until the end of fermentation (Bely et al., 2008; Barrajón et al., 2009; Tufariello et al., 2021). Recently, research to determine the influence of Lt on the fermentation aroma of the wine has been reported (Petitgonnet et al., 2019; Sgouros et al., 2020). It must be noted that, in the totals of higher alcohols, the concentrations were below the threshold that causes sensory “irritating” aromas, and yet fruity aromas might be perceived (Saberi et al., 2012). Interestingly, the production of 1-propanol was always greater at the end of the fermentations in the inoculations with Lt compared to the other treatments, direct proportion to the amount of lactic acid produced. A possible cause might be the small scale of the fermentations, as it has been reported that this compound seems to decrease on a larger scale (Gobbi et al., 2013; Vaquero et al., 2020). Concentrations of the carbonylic compounds, especially diacetyl, which might contribute to buttery sensory notes, were below the perception threshold (Rogerson et al., 2001).

In reference to esters, it has been demonstrated that their production is closely related to the amount of yeast assimilated nitrogen (YAN) available in the must but, depending on the yeast characteristics, their production might increase or decrease with increased levels of YAN (Carrau et al., 2008). The levels of ethyl acetate, with pleasant fruity aromas when the concentration is under 60 mg/L (Medina-Trujillo et al., 2016) or varnish aromas when the concentration is higher, were similar to those observed in other recently published articles in which Lt was also used, with an ethyl acetate production of about 15–25 mg/L (Canonico et al., 2019; Vaquero et al., 2020). In contrast, higher concentrations have also been obtained in previous works (Morata et al., 2019b; Vaquero et al., 2020). It should be noted that the sensory threshold of ethyl lactate is 150 mg/L, contributing to sweet, lactic, and fruit aromas (Peinado et al., 2004; Morata et al., 2019b). This value is almost 10 times higher than that reached in these binary and ternary inoculations. Isoamyl acetate (sweet banana and fruit aroma with a hint of ripe essence) was detected in small quantities in all fermentations and isobutyl acetate (sweet banana and tropical fruit aroma) was barely detected in any of the fermentations (Kemal Balikci et al., 2016; Whitener et al., 2016). Although the existence of acetaldehyde was not demonstrated, in the results of all inoculations, it was around 40 mg/L at the end of fermentation, while with the Sc inoculum, it was always greater than this. Therefore, Lt is a lower producer of acetaldehyde, generally always below Sc (Whitener et al., 2017). With fruit aroma and hints of nuts, this compound is produced by some strains that are higher producers of lactic acid than other yeasts (Sánchez-Palomo et al., 2017). The 2-phenylethyl acetate, a compound that, depending on whether the plant or the microorganisms synthesize it, can be made by the pathways of Shikimate, Ehrlich, and phenylethylamine (Martínez-Avila et al., 2018), with a rose petal and violet aroma descriptor (González Álvarez et al., 2011; Loira et al., 2014; Morata et al., 2019c), was reduced at the end of fermentation. Despite this reduction of the compound, sequential inoculation has been shown to increase the final 2-phenylethyl acetate when Hv --> Sc yeasts are used (Zhang et al., 2020).

On the other hand, Td has been found to produce a characteristic volatile compound, the eter 3-ethoxy-1-propanol (Herraiz et al., 1990; Loira et al., 2015), this compound being derived from homoserine through O-acetylhomoserine (Irwin, 1992), whose aroma is described as blackcurrant notes with a perception threshold of 0.1 mg/L (Peinado et al., 2004; Tao and Zhang, 2010). It was greatly reduced at the end of fermentation in the binary inoculation with Td and disappeared in the ternary co-inoculation with Lt + Td. Other studies have shown that micro-oxygenation and skin contact with the grapes considerably increases this aromatic compound (Samappito and Butkhup, 2010; Antón-Díaz et al., 2016).

It should be noted that there is a strong relationship between the pH and the color of a wine, which is greatly influenced by the yeasts used (Vaquero et al., 2020), and that it is very important to control this in warm areas. It is possible that the greater absorbance at 420 nm in the inoculations was due to the low and slow fermentative power of the binary inoculum Mp and the low total acidity of the binary inoculum Td, thereby resulting in low protection against wine oxidation.

Finally, in the wine sensory analysis in general, the quality was appreciated by the tasters, considering the neutral grape variety that was used. The different yeasts had been able to generate such different and complex wines with hardly any defects, compared to the pure culture of *S. cerevisiae*. The tasters noticed a lower tonality due to a pronounced acidity in wines with binary inoculation of Lt and ternary co-inoculation of Lt + Mp, which resulted in a lower evolution of the wine (Vaquero et al., 2020). It was seen that inoculations with Hv obtained higher scores in the body parameter (Medina et al., 2013). The binary inoculation with Td gave high results in intensity and aroma quality, possibly because it was the one with the lowest amount of volatile acidity and ethyl acetate (Azzolini et al., 2015); on the other hand, this same wine obtained the highest values in hue, perhaps because it had one of the highest pH values (Lee et al., 2019).

## Conclusion

The results obtained show the potential of ternary co-inoculations and the interactions between species, producing metabolism diversification that affects lactic acid and flavor compounds formation. It would therefore be very interesting to continue performing trials to evaluate these yeast combinations, applying differential culture media to monitor the real proportions of each strain of a yeast consortium. These co-inoculations of grape musts are characterized by wines with different and higher secondary compounds than the traditional fermentations of non-*Saccharomyces* monocultures in sequential fermentation with *S. cerevisiae*. The use of ternary co-inoculation with *L. thermotolerans* and other non-*Saccharomyces* species facilitates effective acidification with a more complex aromatic profile. The lower pH also protects the color of the wines and helps to reduce SO_2_ levels.

## Data Availability Statement

The raw data supporting the conclusions of this article will be made available by the authors, without undue reservation.

## Author Contributions

CV, IL, and AM: investigation, literature review, writing, and editing. AM and CV: images design. AM: conceptualization. CG, JH, and FC: critical reading. All authors contributed to the article and approved the submitted version.

### Conflict of Interest

The authors declare that the research was conducted in the absence of any commercial or financial relationships that could be construed as a potential conflict of interest.
